# Prevalence of Self-Reported Attention-Deficit/Hyperactivity Disorder Symptoms and Factors Affecting Attention Span Among Medical Students: A Cross-Sectional Study

**DOI:** 10.7759/cureus.95911

**Published:** 2025-11-01

**Authors:** Shastri Motilal, Aruna Chotak, Farishtah Hoosaney, Emily Kistow, Shayn Ramlogan, Rishna Ramsingh, Shirmel Sankar, Virenda Singh, Rayanne Nagapen

**Affiliations:** 1 Department of Public Health and Primary Care, The University of the West Indies, St. Augustine Campus, St. Augustine, TTO

**Keywords:** anxiety, attention-deficit hyperactivity disorder, depression, medical students, sleep

## Abstract

Objectives

Medical students are a high-risk group for mental health conditions. There is a paucity of research within Caribbean medical schools on attention-deficit/hyperactivity disorder (ADHD), a condition estimated to affect approximately one in 20 adults. The purpose of this research was to determine the prevalence of ADHD-related symptoms and their associated factors affecting attention span among medical students.

Methods

A cross-sectional survey was conducted among a conveniently selected group of currently enrolled medical students in the Department of Public Health and Primary Care, Faculty of Medical Sciences (FMS) of The University of the West Indies, St. Augustine Campus, in Trinidad and Tobago, between January and June 2024. Participants completed an online questionnaire. The Adult ADHD Self-Report Scale, Pittsburgh Sleep Quality Index, Generalized Anxiety Disorder-7, and Patient Health Questionnaire-9 were used to assess ADHD, sleep quality, anxiety, and depression, respectively. Attention spans and lifestyle factors influencing attention were also evaluated. Statistical analyses were done for predictors of ADHD and factors affecting attention span using logistic regression-derived odds ratios (ORs) with 95% confidence limits.

Results

Out of 231 respondents, the mean age was 22.6 years (SD 2.3), with 72.3% identifying as female. The overall prevalence of a positive ADHD screen was 45%, showing no significant association with age (*P* = 0.148) or gender (*P* = 0.788). ADHD positivity was highest in Year 3 (67.3%), significantly greater than Year 1 (OR 3.66, 95% CI 1.62-8.29, *P* = 0.002). High rates of poor sleep quality (82.3%), anxiety (65.4%), and depression (73.2%) were observed, all of which were positively correlated with ADHD (*P* ≤ 0.001). The top five factors affecting attention span included tiredness/energy level (91.3%), academic load (80.1%), sleep quality (79.2%), interest in the subject (78.4%), and time of day (78.4%). Students with ADHD symptoms reported higher levels of exercise, social media usage, caffeine consumption, breakfast skipping, and fluid intake compared to non-ADHD peers (*P* < 0.05 for all).

Conclusions

The study highlights a high prevalence of ADHD-related symptoms and mental health comorbidities among medical students, emphasizing the need for curriculum review and enhanced support services.

## Introduction

Attention-deficit/hyperactivity disorder (ADHD) is a behavioral disorder with symptoms of hyperactivity, impulsivity, and inattention, as declared by the National Institute for Health and Care Excellence [1]. These symptoms often manifest before age 12 and can persist into adulthood, affecting academic, occupational, and social functioning [1]. A systematic review estimated the global prevalence of ADHD at 5.3%, noting variability due to methodological differences across studies [2].

Medical school presents a high-risk environment for students with ADHD. Studies have found the prevalence of ADHD in college and university students to be quite broad, ranging from 2% to 37% [3-6]. Such a wide variation reflects the methods of assessments (brief screening tools vs. clinical interview). Some authors have also suggested adjunctive assessments to complement the interview, which can improve accuracy in diagnosis [7]. In a qualitative study of medical students with ADHD, participants described bullying, isolation, and pressure to conform during clinical placements and exams [8]. Fear of disclosing their diagnosis, driven by toxic competitiveness and concerns over professionalism, led many to mask their symptoms [8]. Untreated ADHD can impair academic progression, clinical performance, and well-being, underscoring the need for greater recognition and sustained support throughout medical training and practice [9].

Attention span, or span of apprehension, has been defined as the maximum number of stimuli or units of information that can be processed at one time after brief presentation [10]. It is directly responsible for determining short-term memory capacity [11]. Various factors influence attention span, including the mental disorder ADHD, internal factors, and the environment [12-16].

Internal factors can both enhance and impair attention span. For instance, chewing gum, consistent practice of meditation, and regular aerobic exercise have all been shown to improve sustained attention and the ability to maintain focus over time [17,18]. By contrast, dehydration, insufficient sleep, or poor sleep quality can significantly reduce one’s capacity to concentrate, leading to difficulties in maintaining attention in settings such as the classroom [19,20]. The relationship between sleep and ADHD, however, is bidirectional: while sleep deprivation can exacerbate inattention and hyperactivity, individuals with ADHD are also more likely to experience sleep-onset difficulties, fragmented sleep, and circadian rhythm disturbances, all of which further impair attention and daytime functioning [21-23]. ADHD and impaired attention span can, in turn, substantially affect academic outcomes. College students with ADHD consistently demonstrate poorer academic achievement, including lower GPA and standardized test scores, as well as higher course withdrawal rates compared with peers without ADHD [24]. Meta-analytic findings confirm that ADHD is associated with large, statistically significant deficits across reading, math, and spelling achievement domains (sample weighted d = 0.71; p = 0.001) and that these effects persist into adulthood [25]. Furthermore, studies using objective performance measures show that adults with ADHD exhibit greater variability in response times and attention lapses than non-ADHD peers, reflecting the sustained attention difficulties underlying their academic challenges [26].

Similarly, external conditions can also exert a strong influence: the physical characteristics of the classroom environment-such as lighting, background noise, or seating arrangement, and the lecturer’s teaching style, including clarity of presentation, pacing, and effective use of multimedia, can either support or undermine students’ attention [27,28].

ADHD frequently co-occurs with mood and anxiety disorders, which can further complicate attention and learning. In the National Comorbidity Survey Replication, Kessler [29] reported that adults with ADHD had markedly elevated odds of comorbid mood disorders (odds ratio (OR) = 5.0) and anxiety disorders (OR = 3.7), underscoring the high psychiatric burden associated with the condition. Similarly, a meta-analysis demonstrated a significant positive association between ADHD and unipolar depression in children and adolescents, with greater co-occurrence observed in clinic-referred samples and when ADHD was diagnosed using DSM-III-R or DSM-IV criteria [30]. Importantly, youth with both ADHD and depression experience greater functional impairment than those with either disorder alone, prompting recommendations for routine screening for depressive symptoms and early, integrated interventions in individuals with ADHD [30].

In clinical settings, medical students are required to engage with patients, actively listen to their concerns, and compile accurate medical histories. Attention span is vital for this level of focus and communication to be effective [31,32]. Existing data highlight the declining attention spans of learners, particularly in the digital age [33-35]. Several studies indicate many factors that influence this decline, including the learning environment, teacher, sleep deprivation, personal health and welfare, and digital distractions [28,34,36-38]. In addition, lengthy lectures are often linked to a decreased attention span in educational settings [38].

Despite these concerns, there is insufficient local and regional research on ADHD and the factors affecting attention span in the Caribbean’s medical education setting. Medical schools in this region serve a diverse student body drawn from multiple island nations with varied sociocultural contexts. High levels of psychological distress have been documented among medical students in Trinidad and Tobago, with studies reporting burnout in over half of students and depressive symptoms in approximately 40% [39]. Subsequent research found anxiety affecting 63%, depression 51%, and stress 48% of medical students [40], while related studies among health professions students identified communication problems, family issues, and feelings of inferiority as significant predictors of anxiety and depression [41]. These findings indicate a persistent burden of anxiety, depression, and burnout in local student populations, supporting the need to assess ADHD symptoms that may overlap with or be masked by these conditions. Without locally generated evidence, educators cannot develop or implement targeted treatment, support programs, or curriculum adaptations to help our students succeed. The objectives of this study were to explore the prevalence of ADHD and determine associations with sleep, anxiety, depression, and lifestyle factors, as well as to examine medical school-specific influences on attention.

## Materials and methods

Study design, setting, and sampling

This study was designed as a quantitative cross-sectional study, which was conducted at the Department of Public Health and Primary Care, Faculty of Medical Sciences (FMS) of The University of the West Indies, St. Augustine Campus, in Trinidad and Tobago. A self-administered online questionnaire (see Appendix) was administered to first- through fifth-year students enrolled in the MBBS program and completed during the period January 2024 to June 2024. The various details of the questionnaire are given in the *Measures* section below. This study was based on a sample of the enrolled MBBS student population (1,300 students) at the FMS.

Sample size calculation

Based on an expected proportion of 24% of ADHD in medical students [5], a minimum of 231 students were required for the survey. This ensured proportions with 95% confidence limits and 5% precision were estimated and adjusted for the finite population size of 1,300.

Inclusion and exclusion criteria

Preclinical students, those enrolled in Years 1-3, and clinical students, those in Years 4-5 of MBBS, were included in the study. Each participant must be at least 18 years old. Students from other schools were not included in this study.

Ethical considerations

Student confidentiality was ensured by collecting anonymized information on a secure device. All students were made aware of the psychological and health support services available. Ethics approval was granted by the University of the West Indies, St. Augustine Campus, Research Ethics Committee (Ref. CREC-SA.2421/11/2023). All participants gave informed consent in accordance with the Declaration of Helsinki.

Data collection

Primary data were gathered (January-June 2024) via an easily interpretable questionnaire that was distributed electronically via WhatsApp groups among the respective MBBS year groups for virtual recruitment and a QR code for in-person recruitment. A convenience sampling approach was employed, as participants self-selected to complete the survey. A consent form was attached to the online survey, and willingness to complete the survey was confirmed by checking the “I agree” button.

Measures

Section 1 defined the basic demographics of age, gender, year of enrollment, and employment status. Section 2 quantified ADHD symptoms with the six-item screener Adult ADHD Self-Report Screening Scale for DSM-5 (ASRS) score of 14 or more, indicating ADHD (91.4% sensitivity, 96.0% specificity) with a 0-24 range [42]. Section 3 addressed attention span in various learning environments. It assigned an ordinal numerical rating score of 1 (extremely difficult to focus) to 10 (very easy to focus) to several learning environments, with the option of not applicable if the student has not experienced a given learning modality. Section 4 asked students to rate several factors that can affect attention span on an ordinal scale ranging from not at all (0) to extremely (4). Section 5 measured depression using the Patient Health Questionnaire 9-item (PHQ9) with a cut-off of 10 or greater (range 0-27), indicating depressive symptomatology (88% sensitivity, 88% specificity) [43]. Section 5 also employed the Generalized Anxiety Disorder 7-item (GAD7) questionnaire, which measured anxiety using a cut-off of 10 or greater (range 0-21), indicating generalized anxiety disorder symptomatology (89% sensitivity, 82% specificity) [44]. Section 6 measured sleep using the Pittsburgh Sleep Quality Index, where a score >5 (range 0-21) was suggestive of poor sleep (sensitivity of 89.6% and specificity of 86.5%) [45]. Section 7 measured various lifestyle factors that can impact attention, measured in minutes or hours of the activity, and frequencies of substance use. The tools in Sections 2, 5, and 6 have all been previously validated. The other sections were created with face validation from the supervisor, with input from students, and pre-testing on 10 students (advised not to participate in the final survey) for feedback. Minor revisions to phrasing and the addition of lifestyle factors and learning environments were the result of student feedback.

Data analysis

Descriptive statistics and the IBM SPSS Statistics, version 25.0 (released 2017, IBM Corp., Armonk, NY) for statistical analysis were used to analyze the collected data. Descriptive data were presented using proportions and means. Comparisons across the data involved Chi-square testing for categorical and ordinal variables and t-testing for continuous data. For binary outcomes, logistic regression was used with demographic and student characteristics as independent variables. Internal consistency was assessed using McDonald’s omega in R studio software. Statistical significance was determined by a P value < 0.05.

## Results

Demographics

A total of 231 responses were received, with the demographics in Table 1. 

**Table 1:** Demographics of the respondents

Variable	Category	n	%
Gender/sex	Male	64	27.7%
	Female	167	72.3%
Ethnicity	African	49	21.2%
	Indo	131	56.7%
	Mixed	45	19.5%
	Other	6	2.6%
Current relationship status	Single	169	73.2%
	In relationship	58	25.1%
	Married/common law	4	1.7%
Year of study	1	50	21.6%
	2	65	28.1%
	3	52	22.5%
	4	43	18.6%
	5	21	9.1%
Currently employed	No	209	90.5%
	Yes	22	9.5%

The average age of the respondents was 22.6 years (SD 2.3). The majority were female (72.3%), of Indo ethnicity (56.7%), single (73.2%), and not employed (90.5%); second-year students were most common (28.1%), followed by third (22.5%), first (21.6%), fourth (18.6%), and fifth years (9.1%).

Attention-deficit/hyperactivity disorder

The mean ASRS score was 12.8 (SD 4.83), with 45% of the sample screening positive for ADHD. The internal consistency (omega (ω)) was 0.874. As shown in Table 2, there were no significant associations between gender, age, ethnicity, relationship, or employment status and ADHD (P > 0.05 for all).

**Table 2:** Association between attention-deficit/hyperactivity disorder (ADHD) and demographics ^#^Percentages calculated as row percentages for each variable’s strata

Variable (n = 231 unless otherwise specified)	ADHD negative^#^	ADHD positive^#^	P-value
Age years, (Mean ± SD)	22.4 ± 2.69	22.8 ± 1.78	0.148
Gender/sex			0.788
Male	34 (53.1%)	30 (46.9%)	
Female	92 (55.1%)	75 (44.9%)	
Ethnicity, n = 227			0.914
African	25 (51.0%)	24 (49.0%)	
East Indian	74 (56.5%)	57 (43.5%)	
Mixed	24 (53.3%)	21 (46.7%)	
Other	3 (50.0%)	3 (50.0%)	
Current relationship status			0.645
Single	93 (55.0%)	76 (45.0%)	
In relationship	30 (51.7%)	28 (48.3%)	
Married/common law	3 (75.0%)	1 (25.0%)	
Year of study, n = 204			0.005
1	32 (64.0%)	18 (36.0%)	
2	40 (61.5%)	25 (38.5%)	
3	17 (32.7%)	35 (67.3%)	
4	27 (62.8%)	16 (37.2%)	
5	10 (47.6%)	11 (52.4%)	
Currently employed			0.653
No	115 (55.0%)	94 (45.0%)	
Yes	11 (50.0%)	11 (50.0%)	

There was a significant association, however, between year of study and ADHD. Year 3 students had the highest rate of 67.3% versus the lowest rate of 36% in Year 1 students. This association was statistically significant with an OR of 3.66 (95% CI 1.62-8.29, P = 0.002).

Anxiety, depression, and sleep quality

Among the respondents (n = 231), the mean total scores were 14.07 ± 6.95 for the PHQ-9, 11.98 ± 6.15 for the GAD-7, and 8.97 ± 3.54 for the PSQI. McDonald’s omega coefficients indicated excellent internal consistency for all scales: PHQ-9 (ω = 0.95), GAD-7 (ω = 0.97), and PSQI (ω = 0.88). The prevalences of screen-positive anxiety, depression, and poor sleep in this study were 65.4%, 73.2%, and 82.3% respectively. Table 3 shows the association between these factors and ADHD.

**Table 3:** Association between attention-deficit/hyperactivity disorder (ADHD) and anxiety, depression, and sleep ^#^Adjusted for year of study

Condition	OR (95% CI) for ADHD	P-value	Adjusted OR^#^ (95% CI) for ADHD	P-value
Poor sleep quality	4.302 (1.889-9.799)	0.001	3.966 (1.711-9.192)	0.001
Depression	13.200 (5.389-32.332)	<0.001	13.350 (5.333-33.416)	<0.001
Anxiety	8.037 (4.080-15.832)	<0.001	8.023 (3.972-16.204)	<0.001

They were all positively associated with ADHD even after adjustment for the year of study. In a subgroup analysis, those with a positive depression and anxiety screen were excluded. The prevalence of a positive ASRS screen in this subgroup was 9.3% (5/54)

Attention span in various learning environments

Students were asked to rate their difficulty in maintaining focus during the range of learning activities they had experienced. Table 4 ranks these activities by the proportion of respondents who rated some level of difficulty maintaining focus in the experienced learning activity. Most students reported difficulty maintaining focus during lectures and preclinical lab sessions, with over 90% affected. Problem-based learning also posed challenges for more than 80% of students. In clinical settings, focus was harder to maintain during ward rounds (71.1%) and while scrubbed in for surgery (64.5%), compared to outpatient clinics (58.2%) and skills labs (58.6%). Sessions on professionalism and ethics (53.3%) and time in the mortuary (49.1%) were associated with the least reported difficulty.

**Table 4:** Students’ difficulty in maintaining focus during different learning activities/environments

Difficulty in maintaining focus by learning activity:	n (%)
Lecture, n = 230	213 (92.6%)
Preclinical lab, n = 224	202 (90.2%)
Problem-based learning, n = 224	181 (80.8%)
Ward rounds, n = 142	101 (71.1%)
Skills lab, n = 222	130 (58.6%)
Outpatient clinic, n = 134	78 (58.2%)
Theater/scrubbed in for surgery, n = 110	71 (64.5%)
Professionalism and ethics session, n = 214	114 (53.3%)
Mortuary, n = 106	52 (49.1%)

Factors affecting attention

Students were asked to rate various factors, both internal and external, on how each affected their attention span during learning activities. Table 5 depicts the distribution of these responses.

**Table 5:** Impact of various factors on the attention span of students

	Self-perceived impact on attention span during learning activity				
Factors, n = 231	Not at all	Slightly	Moderately	Significantly	Extremely
Tiredness/energy level	3 (1.3%)	4 (1.7%)	13 (5.6%)	59 (25.5%)	152 (65.8%)
Interest in subject	6 (2.6%)	13 (5.6%)	31 (13.4%)	48 (20.8%)	133 (57.6%)
Sleep	6 (2.6%)	11 (4.8%)	31 (13.4%)	59 (25.5%)	124 (53.7%)
Personal factors	7 (3.0%)	17 (7.4%)	40 (17.3%)	56 (24.2%)	111 (48.1%)
Social media	17 (7.4%)	30 (13.0%)	45 (19.5%)	31 (13.4%)	108 (46.8%)
Mental health	10 (4.3%)	19 (8.2%)	31 (13.4%)	52 (22.5%)	119 (51.5%)
Academic load	4 (1.7%)	10 (4.3%)	32 (13.9%)	64 (27.7%)	121 (52.4%)
Time of day	6 (2.6%)	11 (4.8%)	33 (14.3%)	80 (34.6%)	101 (43.7%)
Classroom environment	2 (0.9%)	18 (7.8%)	40 (17.3%)	70 (30.3%)	101 (43.7%)
Multitasking	11 (4.8%)	34 (14.7%)	47 (20.3%)	54 (23.4%)	85 (36.8%)
Diet	16 (6.9%)	44 (19.0%)	38 (16.5%)	51 (22.1%)	82 (35.5%)
Caffeine use	37 (16.0%)	36 (15.6%)	42 (18.2%)	33 (14.3%)	83 (35.9%)
Hydration	17 (7.4%)	42 (18.2%)	51 (22.1%)	46 (19.9%)	75 (32.5%)
Exercise	32 (13.9%)	28 (12.1%)	58 (25.1%)	35 (15.2%)	78 (33.8%)
Meditation	66 (28.6%)	63 (27.3%)	47 (20.3%)	18 (7.8%)	37 (16.0%)

Looking at the factors which had either a significant or extreme impact, 91.3% of the respondents indicated that tiredness or energy level affected their attention span, followed by academic load (80.1%), sleep (79.2%), interest in the subject (78.4%), time of day (78.4%), mental health (74.0%), classroom environment (74.0%), and personal factors (72.3%). Lastly, students were asked about various lifestyle factors in general. Table 6 shows the associations between these factors and having a positive ADHD screen.

**Table 6:** Comparison of key lifestyle factors in students with ADHD and students without ADHD ^#^Reported as median (Q1, Q3)

Thinking about the past month, on average how many:	No ADHD^#^	ADHD^#^	p-value
HOURS spent exercising each week?	2.0 (0.0, 5.0)	4.0 (1.5, 6.0)	0.017
HOURS spent on social media each day?	4.0 (3.0, 6.0)	6.0 (4.0, 7.0)	<0.001
CUPS of coffee or caffeinated beverages each day?	1.0 (0.0, 1.0)	1.0 (0.0, 2.0)	0.005
HOURS spent in meditation or mindfulness each day?	0.0 (0.0, 0.0)	0.0 (0.0, 1.0)	0.403
ALCOHOLIC DRINKS had each week?	0.0 (0.0, 1.0)	0.0 (0.0, 1.0)	0.952
TIMES smoked each week?	0.0 (0.0, 0.0)	0.0 (0.0, 0.0)	0.110
DAYS skipped breakfast each week?	2.0 (0.0, 4.0)	3.0 (2.0, 5.0)	0.013
LITERS of fluid consumed each day?	1.5 (0.75, 2.0)	2.0 (2.0, 3.0)	<0.001

As seen, those who screened positive for ADHD were more likely to report exercising, spending time on social media, drinking caffeinated beverages, skipping breakfast, and drinking fluids compared to those without a positive ADHD screen.

## Discussion

This study found an ADHD screen-positive prevalence of 45%, higher than previously reported rates in medical students, which ranged from 11% to 37% [3,45,46]. Academic stress, sleep deprivation, and cultural attitudes toward attention-related behaviors may contribute to this elevated rate. Academic stress can heighten the subjective reporting of ADHD symptoms; one study found that students under stress reported more ADHD-like features even without objective deficits [47]. Sleep deprivation, common among university students, can likewise exaggerate such symptoms. A review demonstrated that inadequate sleep may mimic or exacerbate ADHD manifestations, such as inattention and impulsivity, thereby inflating self-reported symptoms in sleep-deprived individuals [48].

The ASRS tool used in this study has been shown to overestimate ADHD by capturing impulsive traits not specific to the disorder [49]. Population studies indicate that the ASRS can classify 17-26% of adults as probable ADHD, seven to ten times higher than expected, due to its high sensitivity but modest specificity [49]. Similar results in clinical samples suggest that co-existing anxiety or depressive symptoms can amplify self-reported ADHD complaints [50], further increasing false positives [51]. In this study, when participants screening positive for anxiety or depression were excluded, the ASRS-positive prevalence fell sharply to 9%. Hence, ASRS results should be viewed as a sensitive screening indicator rather than diagnostic confirmation; the 45% screen-positive rate more likely reflects symptom endorsement than true disorder prevalence.

There were no associations with gender and ADHD in this study, findings consistent with other studies in the medical education settings [52,53]. Despite the gender similarity, some authors have reported that adult females were more likely to report experiencing inattentive and hyperactive/impulsive symptoms, whereas males more frequently recalled having these symptoms during childhood [54]. This distinction may warrant further study, particularly given the female majority in the student population at our setting.

Due to the COVID-19 epidemic, the third-year students of this study had to complete their first year of medical school online. Transitioning back to face-to-face learning was abrupt and challenging. A Japanese study confirmed that 6-21% of students rated their ADHD symptoms as worsening during the pandemic, which had continued to lead to deteriorations in students’ academic and daily lives in about 10% [55]. Another study found nearly half of students cited difficulty commuting, while others noted increased team projects and anxiety as key negative aspects of returning to in-person classes [56]. Year 3 at the medical school of the author includes five six-credit courses and new pathology subjects, adding more coursework and introducing progressive disclosure questions. This year also incorporates clerkships into weekly plans, significantly increasing the workload compared to earlier years. A descriptive study found that academic stress was also associated with increased depressive symptoms, and attention-related problems contributed to this relationship, suggesting a positive correlation between adult ADHD scores and depression scores with high academic stress [57].

The majority of students in this study reported difficulty maintaining focus during lectures. In a 2012 study of first-year medical students, 62% complained of difficulty concentrating during and following lectures, with 50% stating they needed to be made aware of their learning objectives after the lecture [58]. Furthermore, these students were dissatisfied with passive, lecture-based teaching and preferred active learning methods. Students in our setting seem to prefer active engagement, as seen in more interactive formats. A 2021 study examined how the quality of learning environments influences students’ concentration and performance, rather than the type of learning environment. It found that at least a third to two-thirds of respondents linked sources of stress and poor performance to uncomfortable classroom seating, overcrowded classrooms, unclean classrooms, and poor ventilation, respectively [59].

In the present study, both internal and external factors impacted student concentration. Tiredness or energy level affected the majority of respondents' attention spans, and most cited sleep quality as a major factor. External factors like the classroom environment affected 74.0%. A 2017 study showed that long lectures, off-topic conversations, and lack of sleep significantly disrupted concentration [60]. Le (2021) categorized factors into internal (e.g., interest, sickness, hunger) and external (e.g., classroom environment, teaching methods), noting that tiredness and subject interest as key [19]. Other studies have similarly emphasized the importance of varied teaching techniques and a conducive classroom environment [61,62].

In this study, students with ADHD symptoms spent significantly more time on social media. This aligns with earlier studies linking social media use to shorter attention spans and greater psychological distress [63-65]. In our study, ADHD screen-positive students were more likely to use exercise, caffeine, and hydration, factors that may help manage attention. A 2014 review found that exercise reduced symptoms of ADHD and led to improvements in social behavior, motor skills, and neurophysiological parameters [66]. In addition, another review noted that aerobic exercise significantly improved attention, hyperactivity, impulsivity, anxiety, and executive function in children with ADHD [67]. ADHD symptoms, such as inattention and impulsivity, can result from eating habits. According to a prospective study, Western eating behavior was associated with ADHD [68]. A Chinese study that examined the link between breakfast skipping and ADHD suggested a causal relationship [69]. Lastly, caffeine is widely recognized as a CNS stimulant for temporarily heightening alertness and cognitive function, which can be beneficial to individuals with ADHD seeking to improve attention and concentration. A 2020 study concluded that there is a positive influence of caffeine and caffeinated compounds at variable degrees on cognition and impulsive behavior [70].

Recommendations

The high prevalence of ADHD symptoms and the availability of the brief ASRS tool begs the question: should we screen for ADHD in at-risk groups? Some authors have advocated for screening among medical students [71]. However, further research is needed to validate the ADHD screening tool used and clinical diagnoses, given its reduced specificity, especially in samples with comorbid anxiety and depression. Given the difficulties with maintaining focus in the many learning environments, a curriculum review is warranted to examine how teaching is delivered. Interventions such as a blended learning environment of both practical and non-practical skills should be implemented for students [72]. The way lectures are delivered can also enhance attention span. Harmonized strategies among lecturers to improve student engagement could improve attention [37]. The study should be done using a more representative sample. Repeat surveys for trends will also allow the faculty to monitor the prevalence of ADHD and its comorbidities. Educational and coping strategies to improve sleep, self-care, and reduce social media usage are also needed [35,73,74].

This study employed validated and reliable measures of sleep, depression, anxiety, and ADHD-related symptoms, ensuring methodological rigor. It is the first to describe ADHD symptom patterns and attention-span-related factors among medical students in a Caribbean medical school context, providing culturally relevant baseline data for the region. By assessing attention span across unique medical school learning environments, such as clinical rotations, laboratories, and problem-based learning sessions, the study offers novel insight into how instructional settings may influence concentration and engagement. Examining multiple psychological domains together allowed a holistic view of student well-being and cognitive functioning. There were, however, several limitations of this work. Self-reporting bias (e.g., recall, social desirability) may have overestimated the prevalence of ADHD symptoms. The cut-offs used for a positive ASRS screen were not validated in the authors' setting and may also have contributed to the high reported burden of ADHD symptoms. The convenient nature of this online survey meant it was possible that only students who have symptoms may have been drawn to participate, also inflating the prevalence of ADHD symptoms. This study did not exclude participants based on anxiety or depressive symptoms or self-reported diagnosis, which may have also confounded the apparently high burden of ADHD symptoms. In addition, the unequal male-female ratio (72.3% females) suggests a potential response bias, as female students may have been more likely to complete or disclose symptoms on self-report questionnaires, limiting the representativeness of the sample. These factors limit the generalizability of findings to the student population. The questionnaires used, though well-validated in settings external to the Caribbean, were screening tools and do not provide a medical diagnosis of ADHD, depression, or anxiety disorders. The cross-sectional nature of this study also meant that causal inferences cannot be drawn from the associations found.

## Conclusions

This study highlights a high burden of psychological symptoms among medical students, with substantial proportions screening positive for anxiety, depression, and stress, alongside sleep deprivation and unhealthy lifestyle patterns. While ADHD-like symptoms were prevalent, their overlap with these co-existing mental health issues underscores the complexity of attributing attention difficulties to a single cause. Rather than interpreting such findings as evidence of undiagnosed ADHD, they may reflect the cumulative strain of academic stress, sleep loss, and emotional distress within medical training.

Addressing this burden requires institution-level strategies that promote mental well-being, including early screening, supportive counseling services, and psychoeducation on coping strategies such as stress management, healthy sleep hygiene, and moderated social media use. Re-examining curriculum structure and learning delivery to reduce excessive workload and encourage interactive, student-centered learning may further support concentration and resilience among medical students.

## Figures and Tables

**Figure 1:**
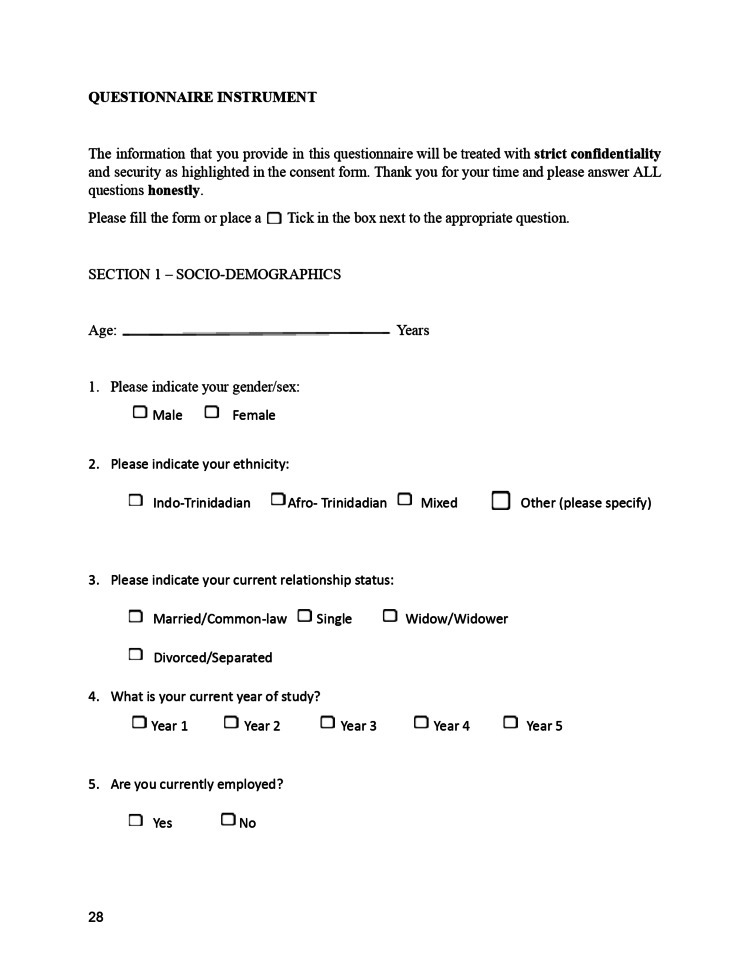
Section 1: sociodemographics

**Figure 2:**
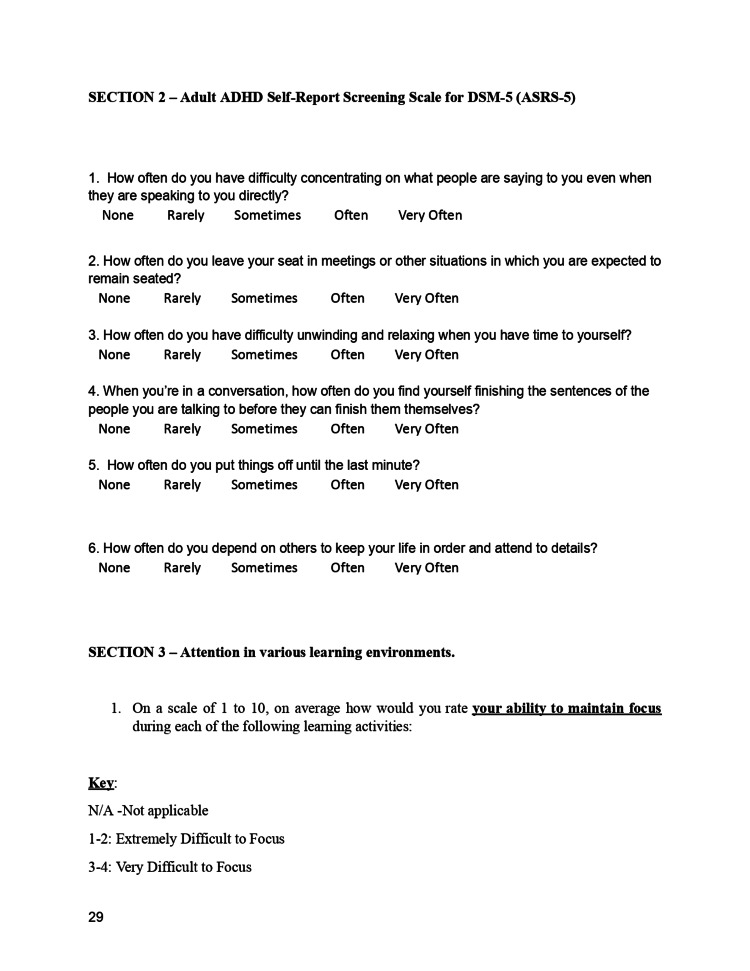
Section 2: Adult ADHD Self-Report Screening Scale for DSM-5 (ASRS-5)

**Figure 3:**
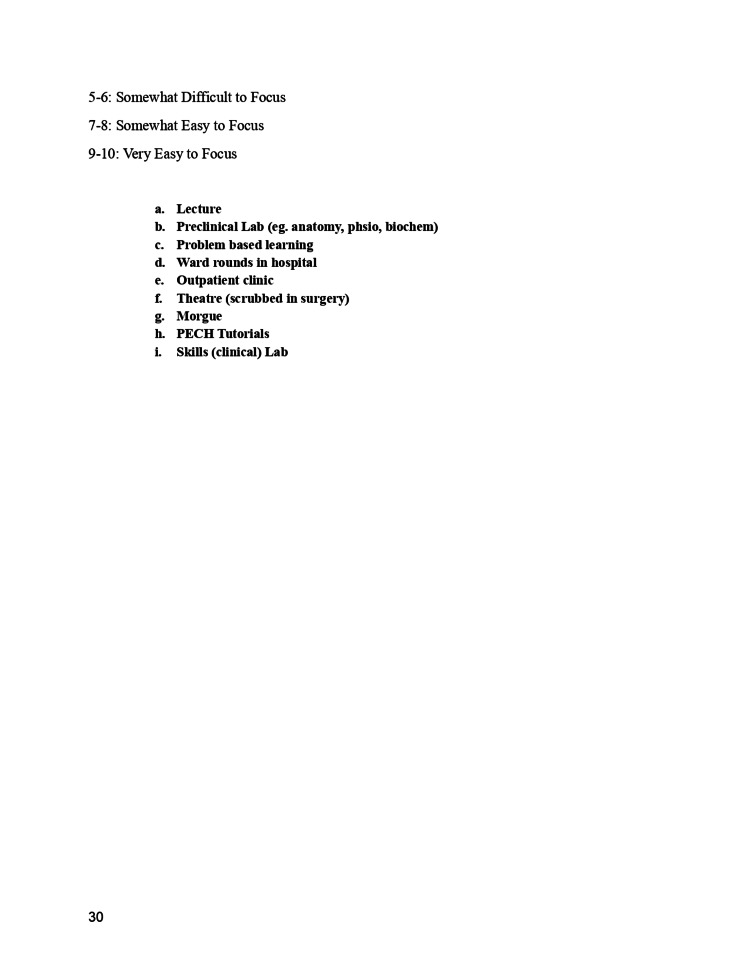
Section 3: attention in various learning environments (cont.)

**Figure 4:**
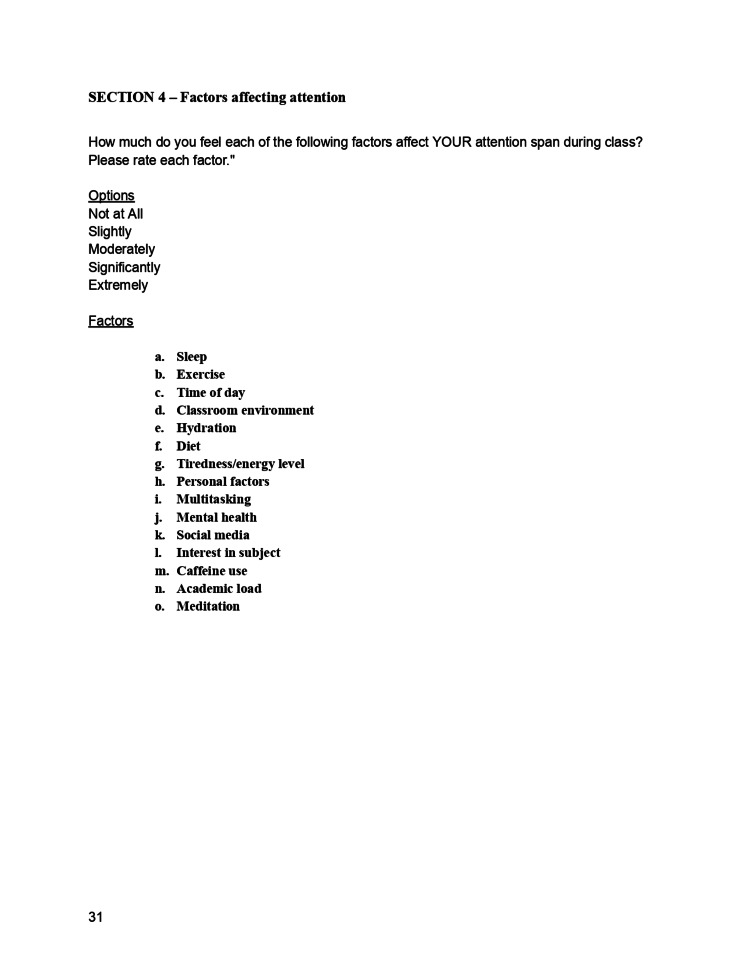
Section 4: factors affecting attention

**Figure 5:**
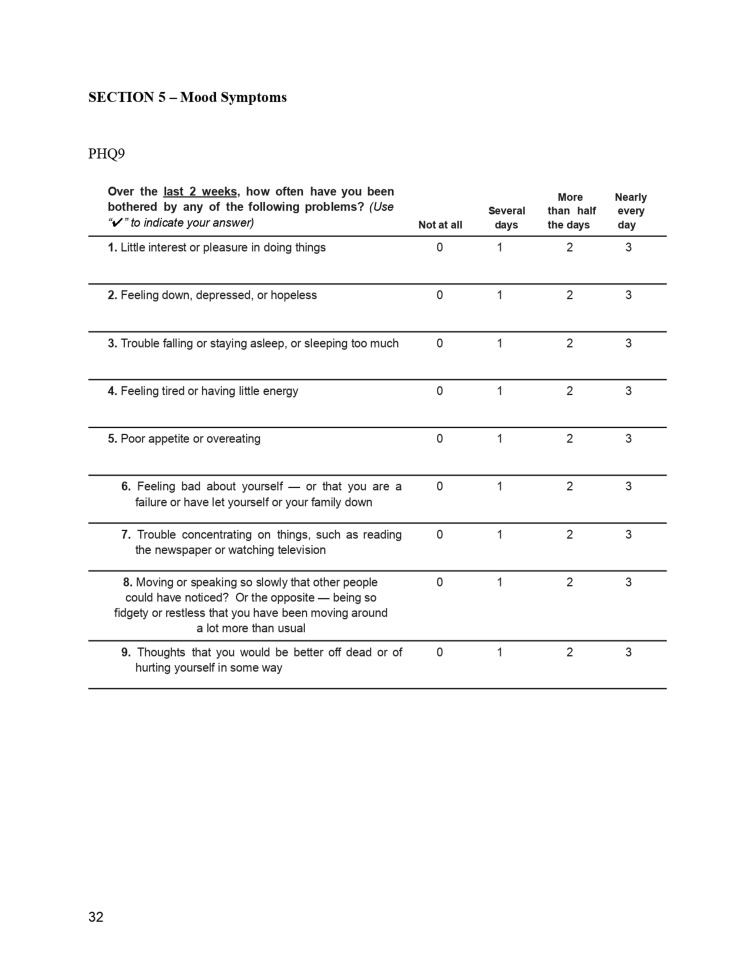
Section 5: mood symptoms

**Figure 6:**
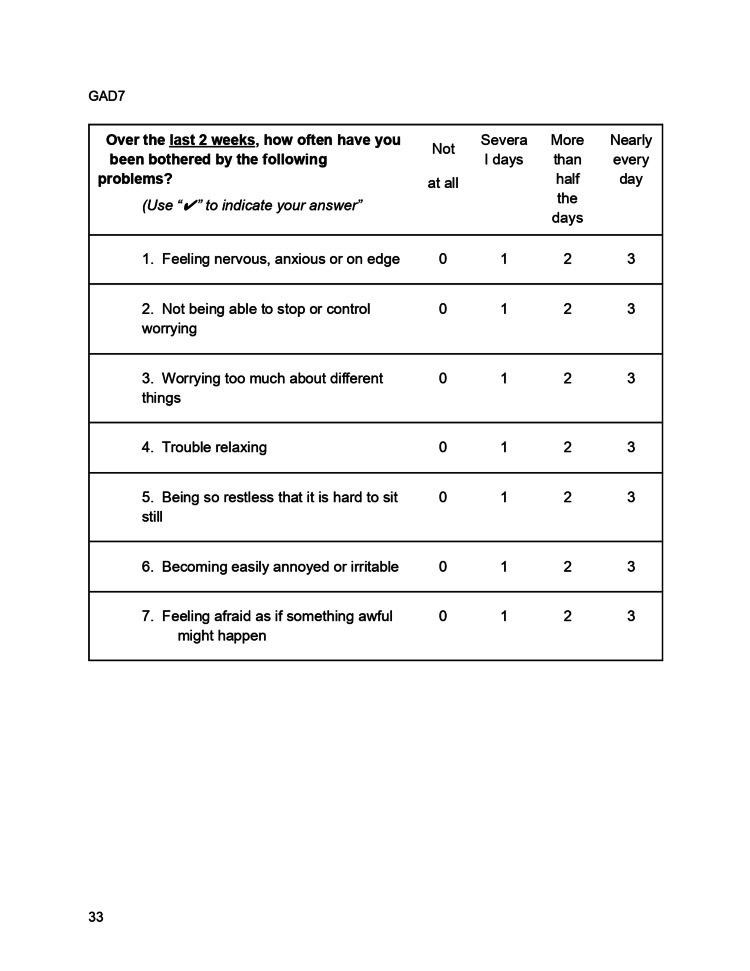
Section 5 (cont.)

**Figure 7:**
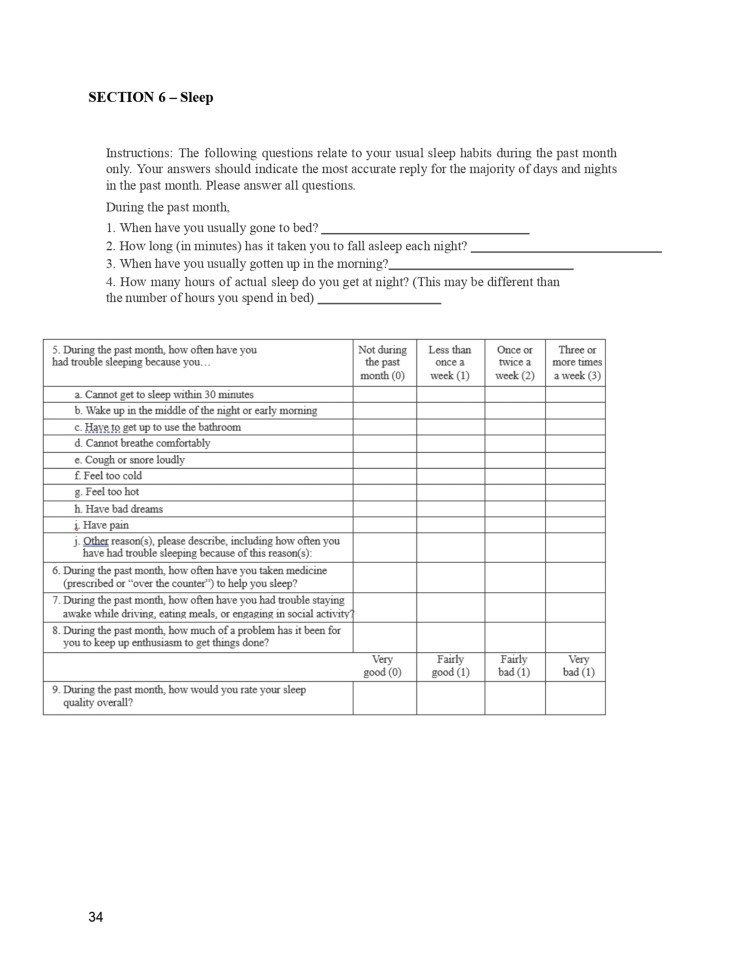
Section 6: sleep

**Figure 8:**
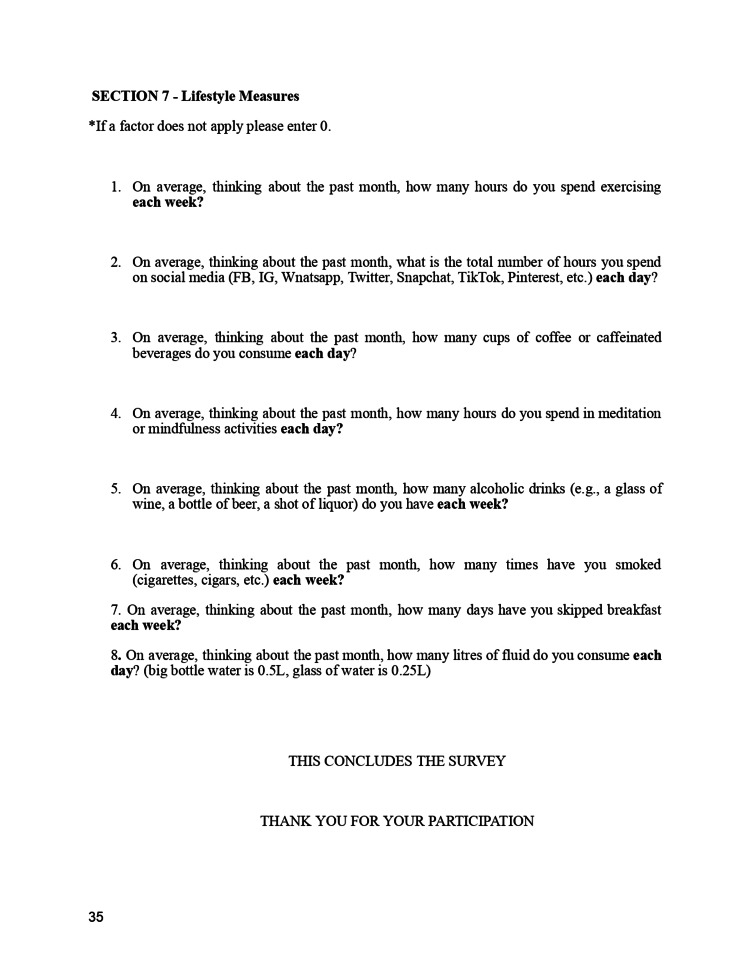
Section 7: lifestyle measures

## Appendices

Study questionnaire

## References

[1] NICE NICE (2024). NICE: attention deficit hyperactivity disorder: diagnosis and management. https://www.nice.org.uk/guidance/ng87.

[2] Polanczyk G, de Lima MS, Horta BL, Biederman J, Rohde LA (2007). The worldwide prevalence of ADHD: a systematic review and metaregression analysis. Am J Psychiatry.

[3] Mattos P, Nazar BP, Tannock R (2018). By the book: ADHD prevalence in medical students varies with analogous methods of addressing DSM items. Braz J Psychiatry.

[4] Pagespetit È, Pagerols M, Barrés N (2025). ADHD and academic performance in college students: a systematic review. J Atten Disord.

[5] Hotez E, Rosenau KA, Fernandes P, Eagan K, Shea L, Kuo AA (2022). A national cross-sectional study of the characteristics, strengths, and challenges of college students with attention deficit hyperactivity disorder. Cureus.

[6] Nugent K, Smart W (2014). Attention-deficit/hyperactivity disorder in postsecondary students. Neuropsychiatr Dis Treat.

[7] Marshall P, Hoelzle J, Nikolas M (2021). Diagnosing attention-deficit/hyperactivity disorder (ADHD) in young adults: a qualitative review of the utility of assessment measures and recommendations for improving the diagnostic process. Clin Neuropsychol.

[8] Godfrey-Harris M, Shaw SC (2023). The experiences of medical students with ADHD: a phenomenological study. PLoS One.

[9] Im DS, Tamarelli CM (2023). Attention deficit hyperactivity disorder in medical learners and physicians and a potentially helpful educational tool. Adv Med Educ Pract.

[10] Cohen R (2011). Span of Apprehension. Encyclopedia of clinical neuropsychology.

[11] Cowan N (2001). The magical number 4 in short-term memory: a reconsideration of mental storage capacity. Behav Brain Sci.

[12] Barkley RA (1997). Behavioral inhibition, sustained attention, and executive functions: constructing a unifying theory of ADHD. Psychol Bull.

[13] Lim J, Dinges DF (2010). A meta-analysis of the impact of short-term sleep deprivation on cognitive variables. Psychol Bull.

[14] Lavie N (2005). Distracted and confused?: selective attention under load. Trends Cogn Sci.

[15] Posner MI, Petersen SE (1990). The attention system of the human brain. Annu Rev Neurosci.

[16] Hirano Y, Onozuka M (2015). Chewing and attention: a positive effect on sustained attention. Biomed Res Int.

[17] Smith PJ, Blumenthal JA, Hoffman BM (2010). Aerobic exercise and neurocognitive performance: a meta-analytic review of randomized controlled trials. Psychosom Med.

[18] Cicekci M, Sadik F (2019). Teachers’ and students’ opinions about students’ attention problems during the lesson. J Educ Learn.

[19] Le HV (2021). An investigation into factors affecting concentration of university students. J Engl Lang Teach Appl Linguist.

[20] Walters AS, Silvestri R, Zucconi M, Chandrashekariah R, Konofal E (2008). Review of the possible relationship and hypothetical links between attention deficit hyperactivity disorder (ADHD) and the simple sleep related movement disorders, parasomnias, hypersomnias, and circadian rhythm disorders. J Clin Sleep Med.

[21] Yoon SY, Jain U, Shapiro C (2012). Sleep in attention-deficit/hyperactivity disorder in children and adults: past, present, and future. Sleep Med Rev.

[22] Becker SP, Langberg JM, Eadeh HM, Isaacson PA, Bourchtein E (2019). Sleep and daytime sleepiness in adolescents with and without ADHD: differences across ratings, daily diary, and actigraphy. J Child Psychol Psychiatry.

[23] Advokat C, Lane SM, Luo C (2011). College students with and without ADHD: comparison of self-report of medication usage, study habits, and academic achievement. J Atten Disord.

[24] Frazier TW, Youngstrom EA, Glutting JJ, Watkins MW (2007). ADHD and achievement: meta-analysis of the child, adolescent, and adult literatures and a concomitant study with college students. J Learn Disabil.

[25] Advokat C, Martino L, Hill BD, Gouvier W (2007). Continuous Performance Test (CPT) of college students with ADHD, psychiatric disorders, cognitive deficits, or no diagnosis. J Atten Disord.

[26] Agravante III J, Inserto F, Nazareno L (2019). Factors affecting the attention span of Bachelor of Science in Nursing students during classroom engagement. Bachelor of Science in Nursing [Internet.

[27] Rahiminia E, Yazdani S, Rahiminia H (2020). Factors Affecting Concentration and Attendance in the Classroom from Students’ Point of View in Qom University of Medical Sciences (2018). Educ Res Med Sci.

[28] Kessler RC, Adler L, Barkley R (2006). The prevalence and correlates of adult ADHD in the United States: results from the National Comorbidity Survey Replication. Am J Psychiatry.

[29] Meinzer MC, Pettit JW, Viswesvaran C (2014). The co-occurrence of attention-deficit/hyperactivity disorder and unipolar depression in children and adolescents: a meta-analytic review. Clin Psychol Rev.

[30] Chen H, Liu C, Zhou F (2022). Focused-attention meditation improves flow, communication skills, and safety attitudes of surgeons. Int J Environ Res Public Health.

[31] Mundy P, Newell L (2007). Attention, joint attention, and social cognition. Curr Dir Psychol Sci.

[32] Oaten J. (2025). Combating the attention span crisis in our students. https://santamaria.wa.edu.au/decreasing-attention-spans-jennifer-oaten/.

[33] Attia NA, Baig L, Marzouk YI, Khan A (2017). The potential effect of technology and distractions on undergraduate students' concentration. Pak J Med Sci.

[34] Lodge JM, Harrison WJ (2019). The role of attention in learning in the digital age. Yale J Biol Med.

[35] Mota Albuquerque P, Ribeiro Franco CM, Sampaio Rocha-Filho PA (2023). Assessing the impact of sleep restriction on the attention and executive functions of medical students: a prospective cohort study. Acta Neurol Belg.

[36] Bahşi J, Ilhan Ç, Çetkin M (2017). Evaluation of attention-motivation level, studying environment and methods of medical faculty students. Eur J Ther.

[37] Bradbury NA (2016). Attention span during lectures: 8 seconds, 10 minutes, or more?. Adv Physiol Educ.

[38] Youssef FF (2016). Medical student stress, burnout and depression in Trinidad and Tobago. Acad Psychiatry.

[39] Sahu PK, Nayak BS, Rodrigues V, Umakanthan S (2020). Prevalence of psychological distress among undergraduate medical students: a cross-sectional study. Int J Appl Basic Med Res.

[40] Nayak BS, Sahu PK (2022). Socio-demographic and educational factors associated with depression, anxiety and stress among health professions students. Psychol Health Med.

[41] Ustun B, Adler LA, Rudin C (2017). The World Health Organization Adult Attention-Deficit/Hyperactivity Disorder Self-Report Screening Scale for DSM-5. JAMA Psychiatry.

[42] Kroenke K, Spitzer RL, Williams JB (2001). The PHQ-9: validity of a brief depression severity measure. J Gen Intern Med.

[43] Spitzer RL, Kroenke K, Williams JB, Löwe B (2006). A brief measure for assessing generalized anxiety disorder: the GAD-7. Arch Intern Med.

[44] Buysse DJ, Reynolds CF, Monk TH, Berman SR, Kupfer DJ (1989). The Pittsburgh sleep quality index: a new instrument for psychiatric practice and research. Psychiatry Res.

[45] Alrahili N, Aldakheel AA, AlUbied A (2019). Prevalence of adult attention deficit hyperactivity disorder among medical students in Riyadh City. Int J Med Dev Ctries.

[46] Atwoli L, Owiti P, Manguro G, Ndambuki D (2011). Attention deficit hyperactivity disorder symptom self-report among medical students in Eldoret, Kenya. Afr J Psychiatry (Johannesbg).

[47] Akçay E, Akdeniz G, Özışık P, Yılmaz G (2025). Higher perceived stress increases the subjective reporting of ADHD: a sample of medical students. https://www.semanticscholar.org/paper/Higher-Perceived-Stress-Increases-the-Subjective-of-Ak%C3%A7ay-Akdeniz/31cbf97e9055984bd8387639aac9bc7ed7a493e4.

[48] Cassoff J, Wiebe ST, Gruber R (2012). Sleep patterns and the risk for ADHD: a review. Nat Sci Sleep.

[49] Chamberlain SR, Cortese S, Grant JE (2021). Screening for adult ADHD using brief rating tools: what can we conclude from a positive screen? Some caveats. Compr Psychiatry.

[50] Dunlop BW, Wu R, Helms K (2018). Performance of the adult ADHD Self-Report Scale-v1.1 in adults with major depressive disorder. Behav Sci (Basel).

[51] Hines JL, King TS, Curry WJ (2012). The adult ADHD self-report scale for screening for adult attention deficit-hyperactivity disorder (ADHD). J Am Board Fam Med.

[52] Alsafar FA, Alsaad AJ, Albukhaytan WA (2024). Prevalence of adult attention deficit hyperactivity disorder (ADHD) among medical students in the Eastern Province of Saudi Arabia. Saudi Med J.

[53] Shi M, Liu L, Sun X, Wang L (2018). Associations between symptoms of attention-deficit/ hyperactivity disorder and life satisfaction in medical students: the mediating effect of resilience. BMC Med Educ.

[54] Platania NM, Starreveld DE, Wynchank D, Beekman AT, Kooij S (2025). Bias by gender: exploring gender-based differences in the endorsement of ADHD symptoms and impairment among adult patients. Front Glob Womens Health.

[55] Takeda T, Tsuji Y, Akatsu R, Nomura T (2023). Initial impact of the COVID-19 outbreak on ADHD symptoms among university students in Japan. J Korean Acad Child Adolesc Psychiatry.

[56] Teacher TS (2025). Analysis of students’ transition back to face-to-face instruction. scholarlyteacher.

[57] Rabiner DL, Anastopoulos AD, Costello J, Hoyle RH, Swartzwelder HS (2008). Adjustment to college in students with ADHD. J Atten Disord.

[58] Almoallim H, Aldahlawi S, Alqahtani E, Alqurashi S, Munshi A (2012). Difficulties facing first-year medical students at Umm Alqura University in Saudi Arabia. East Mediterr Health J.

[59] Alotiby A, Almaghrabi M, Alosaimy R (2021). Learning environment quality for medical students at Umm Al-Qura University: a comprehensive study on stressors, sources, and solutions after introduction of a new Bachelor of Medicine and Bachelor of Surgery (MBBS) curriculum. Adv Med Educ Pract.

[60] Mohamed A, Alorabi H, Aljutaili H (2017). Medical students perception of factors disrupt their concentration during lectures at King Saud University, Saudi Arabia. Int J Acad Sci Res.

[61] Burke B (2008). Creating communicative classrooms with experiential design. Foreign Lang Ann.

[62] Gerschler J (2025). Oaxaca State University System ESL Conference: slassroom strategies for maintaining student focus. https://www.researchgate.net/profile/Jared-Gerschler/publication/326066230_Classroom_Strategies_for_Maintaining_Student_Focus/links/5b3651ae0f7e9b0df5d99750/Classroom-Strategies-for-Maintaining-Student-Focus.pdf.

[63] Alaparthi K (2025). Technology and digital media’s impact on attention span in teenagers and young adults. Soc Sci Res Net.

[64] Mahalingham T, Howell J, Clarke PJ (2022). Attention control moderates the relationship between social media use and psychological distress. J Affect Disord.

[65] Sahu DP, Taywade M, Malla PS (2024). Screen time among medical and nursing students and its correlation with sleep quality and attention span: a cross-sectional study. Cureus.

[66] Kamp CF, Sperlich B, Holmberg HC (2014). Exercise reduces the symptoms of attention-deficit/hyperactivity disorder and improves social behaviour, motor skills, strength and neuropsychological parameters. Acta Paediatr.

[67] Cerrillo-Urbina AJ, García-Hermoso A, Sánchez-López M, Pardo-Guijarro MJ, Santos Gómez JL, Martínez-Vizcaíno V (2015). The effects of physical exercise in children with attention deficit hyperactivity disorder: a systematic review and meta-analysis of randomized control trials. Child Care Health Dev.

[68] Howard AL, Robinson M, Smith GJ, Ambrosini GL, Piek JP, Oddy WH (2011). ADHD is associated with a "Western" dietary pattern in adolescents. J Atten Disord.

[69] Zhang Z, Tan J, Luo Q (2024). Associations between breakfast skipping and outcomes in neuropsychiatric disorders, cognitive performance, and frailty: a Mendelian randomization study. BMC Psychiatry.

[70] Cipollone G, Gehrman P, Manni C (2020). Exploring the role of caffeine use in adult-ADHD symptom severity of US army soldiers. J Clin Med.

[71] Anbarasan D, Kitchin M, Adler LA (2020). Screening for adult ADHD. Curr Psychiatry Rep.

[72] Çakmakkaya ÖS, Meydanlı EG, Kafadar AM (2024). Factors affecting medical students' satisfaction with online learning: a regression analysis of a survey. BMC Med Educ.

[73] Olson JA, Sandra DA, Chmoulevitch D, Raz A, Veissière SP (2022). A nudge-based intervention to reduce problematic smartphone use: randomised controlled trial. Int J Ment Health Addict.

[74] Yakobi O, Smilek D, Danckert J (2021). The effects of mindfulness meditation on attention, executive control and working memory in healthy adults: a meta-analysis of randomized controlled trials. Cognit Ther Res.

